# Efficient regeneration of mature castanopsis hystrix from *in vitro* stem explants

**DOI:** 10.3389/fpls.2022.914652

**Published:** 2022-08-12

**Authors:** Heng Zhang, Mengqing Guo, Qiaona Wu, Mengqiu Zhao, Ruiping Li, Xiaomei Deng, Ruchun Xi

**Affiliations:** ^1^Guangdong Key Laboratory for Innovative Development and Utilization of Forest Plant Germplasm, Guangzhou, China; ^2^College of Forestry and Landscape Architecture, South China Agricultural University, Guangzhou, China

**Keywords:** *Castanopsis hystrix*, mature tree, disinfection, *in vitro* regeneration, browning, adventitious roots

## Abstract

*Castanopsis hystrix* is one of the main timber trees grown in China. However, severe shortage of natural seeds and the difficulty of explant regeneration has limited seedling supply. As such, there is a need for research on asexual multiplication of *C. hystrix*. This study established a rapid propagation technology system for *C. hystrix* genotypes, including explant treatment, proliferation, and rooting. HZ (a modified MS medium) supplemented with 4.4 μM BA and 0.5 μM IBA was found to be the optimal medium for shoot sprouting. The maximum proliferation coefficient and the number of effective shoots was obtained on HZ medium supplemented with 2.6 μM BA and 1.0 μM IBA, were 3.00 and 5.63, respectively. A rooting rate of 83.33% was achieved using half-strength HZ medium supplemented with 3.2 μM NAA. Adding vitamin C (80 mg⋅l^–1^) for 7 days in a dark environment reduced the browning rate, while increasing the proliferation rate. Additionally, through cytological observation, we established how and where adventitious roots occur. The survival rate of transplanted plantlets was > 90%. This is the first report of an *in vitro* regeneration technique that uses stem segments of mature *C. hystrix* as explants.

## Introduction

*Castanopsis hystrix* belongs to the genus *Castanopsis* of the Fagaceae family, mainly distributed in the tropical and subtropical regions of southern China, and has very important economic, ecological and social value (Su et al., 2004; Wu et al., 2012; Liu et al., 2020; Souma and Ishikawa, 2020). As a native broad-leaved precious timber tree species, its seeds can be used to extract tanning agents and foods rich in tannins and starch (Chen et al., 1993a,b; Tanaka et al., 1993; Chang et al., 1995). Its rapid growth also has significant ecological functions such as water and soil conservation, disaster prevention, and mitigation. Moreover, they play a critical role in biodiversity and the global carbon budget (Xiaofang et al., 2015; You et al., 2018; Liang et al., 2019; Zhang et al., 2019).

Difficulties in breeding have restricted the application of superior genotypes of *C. hystrix*. Currently, seedling production is the primary breeding method. However, seedling production is difficult because of the phenomenon of “biennial fruiting” and difficulties in seed storage. The different genotypes have considerable growth rate, material, and seed yield variations. Moreover, the seedlings are not consistent with the growth characteristics of mother trees (San-José et al., 2010). Although the grafting method has been reported, the survival rate is only 44%, and there is a “partial crown” phenomenon (Baofu et al., 2018). A lack of sufficient branches also limits large-scale production (Ahuja and Jain, 2017).

Biotechnological methods may be valuable in the propagation and conservation of selected genotypes (Gaidamashvili and Benelli, 2021). *In vitro* propagation is not only less affected by the external environment but can also produce orderly and consistent young plants with ideal characteristics (Kumar et al., 2011; Martínez et al., 2021; Chakraborty et al., 2022). Adult trees are difficult to use as material because of the large number of endophytic bacteria, difficulty establishing an aseptic system, and shoot induction and differentiation, which dramatically limits *in vitro* propagation (Martínez et al., 2017a, 2021). Therefore, it is essential to establish an efficient propagation technology for mature *C. hystrix*. In a prior study, explored the technique of using the seeds of mature *C. hystrix* as explants for tissue culture (Ruchun et al., 2016; Chunyan and Suyun, 2017). Research using mature *C. hystrix* branches as a material for *in vitro* culture techniques is limited.

In the present study, we established an efficient and rapid propagation protocol to ensure the fixation of these ideal traits to the next generation. The findings of this study can be used for clonal propagation and germplasm conservation of superior genotypes of *C. hystrix*.

## Materials and methods

### Plant materials and culture conditions

Semi-lignified branches were collected from a miniature grafting nursery (Figure 2B) in the College of Forestry and Landscape Architecture’s greenhouse, South China Agricultural University (113°21 E, 23°09 N). The grafting material was obtained from a mature tree (over 100 years old, Figure 2A), having a favorable genotype. It has the advantages of firmness and high hardness of wood.

**FIGURE 1:**
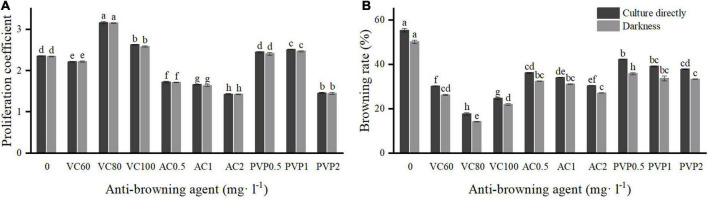
Effects of different types and concentrations of anti-browning agents on shoot proliferation and browning. Group a (left) was cultured directly under light. Group b (right) was transferred to light culture after a 7-day darkness treatment. **(A)** Proliferation coefficient; **(B)** browning rate. Different uppercase letters in the same column indicate a significant difference, as per Duncan’s multiple range test (*p* ≤ 0.05, *n* = 3 indicates three replicates).

**FIGURE 2:**
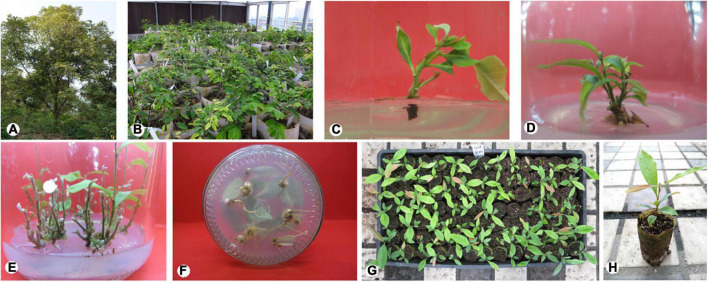
Micropropagation of mature *Castanopsis hystrix* trees using axillary node explants. **(A)** Mother plant; **(B)** the miniature grafting nursery; **(C)** shoot sprouting; **(D,E)** shoot proliferation; **(F)** rooting; **(G)** plantlet acclimatization for 30 days; **(H)** plantlet acclimatization for 90 days.

All culture media were adjusted to pH 5.7, solidified with 6 g⋅l^–1^ agar, and autoclaved at 121°C for 20 min (Corredoira et al., 2006). For the sprouting and proliferation, 30% sucrose was added in the regeneration medium while for root induction 15% sucrose was supplied (Purohit et al., 2002). After inoculation, the plantlets were incubated in a growth chamber with a 12 h/day photoperiod (provided by cool-white fluorescent lamps at 50–60 μmol m^–2^ s^–1^) at 23 ± 2°C (Martínez et al., 2017b).

### Disinfection and shoot sprouting

Branches approximately 5–15 cm in length were collected as the initial material. After two-thirds of the leaves were cut off, they were washed with 5% (v: v) liquid detergent (consisting of surfactant and soft water) solution, rinsed under running water for 1 h, and then moved to a clean bench. Initial materials were cut into stem segments with 1–2 axillary shoots (1–2 cm in length) and then placed into sterile glass bottles according to the degree of lignification (semi-lignified). Thirty explants were placed in each bottle for surface disinfection. A total of 6 treatments of disinfection were used in this study (Table 1). After disinfection, both ends of the wound of explants were cut (0.5–1.5 cm) and inoculated on MS supplemented with 4.4 μM BA and 0.5 μM indole-3-butyric acid (IBA). The contamination, browning, and survival rates of each treatment were recorded after 30 days.

**TABLE 1:** Effects of different disinfection treatments on the survival of explants.

No.	Disinfection treatments					Contamination rate (%) (Mean ± SE; *n* = 3)	Browning rate (%) (Mean ± SE; *n* = 3)	Survival rate (%) (Mean ± SE; *n* = 3)
	NaOCl (2%, min)	Ethanol (75%, s)	Benzalkonium Bromide (1%, min)	Mercuric chloride (0.1%, min)	Tween 80 (drop)			
1	5–7	–	–	–	–	40.00 ± 1.92a	12.22 ± 1.11b	35.56 ± 4.00c
2	–	–	–	1–4	–	31.11 ± 2.22b	13.33 ± 1.92b	42.22 ± 2.22c
3	–	30	–	1–4	–	28.89 ± 1.11b	25.56 ± 2.22a	52.22 ± 4.00b
4	–	–	2–4	1–4	–	21.11 ± 2.94cd	14.44 ± 2.22b	62.22 ± 2.94b
5	–	–	2–4	1–4	2–3	17.78 ± 2.94d	11.11 ± 1.11b	74.44 ± 2.94a
6	–	30	2–4	1–4	–	25.56 ± 2.22bc	27.78 ± 2.22a	55.56 ± 2.94b

The disinfection time of benzalkonium bromide and mercuric chloride depended on the degree of lignification of the explants. After disinfection with disinfectant agent, all explants were rinsed four times with sterile distilled water. Different lowercase letters in the same column indicate significant differences, as per Duncan’s multiple range test (p ≤ 0.05, n = 3 indicates three replicates).

To optimize shoot sprouting, we chose five different basal media containing BA 4.4 μM and IBA 0.5 μM for comparison (Table 2). Among them, HZ is a medium that has been modified from MS. The macronutrient component of the HZ medium is composed of 520 mg⋅l^–1^ NH_4_NO_3_; 950 mg⋅l^–1^ KNO_3_, 495 mg⋅l^–1^ KH_2_PO_4_, 370 mg⋅l^–1^ MgSO_4_⋅7H_2_O, and 440 mg⋅l^–1^ CaCl_2_⋅2H_2_O, other mineral nutrients are the same in composition as in MS. On this basis, to further optimize the shoot sprouting, BA (2.2, 4.4 and 8.8 μM), α-naphthaleneacetic acid (NAA) (0, 0.5 and 2.7 μM), and IBA (0, 0.5 and 2.5 μM) with different combinations were added to medium. Nine treatments were designed based on the L9 (3^4^) orthogonal test (Table 3). Observations were made after 30 days, including shoot sprouting rate, shoot length, and growth state.

**TABLE 2:** Effects of different basal media on initial shoot sprouting.

No.	Basal medium	Time of shoot initiation (d)	Shoot induction rate (%) (Mean ± SE; *n* = 3)	Shoot length (cm) (Mean ± SE; *n* = 3)	Growth state of shoots
1	MS	21–24	8.89 ± 1.11d	0.21 ± 0.03cd	Hyperhydricity, thin shoots
2	1/2MS	18–21	11.11 ± 1.11d	0.17 ± 0.01d	Small and short shoots
3	1/4MS	14–16	42.22 ± 1.11b	0.74 ± 0.01b	Yellow-green and thin
4	HZ	12–14	71.11 ± 2.94a	0.95 ± 0.01a	Robust shoots, fast growth
5	B5	14–16	25.56 ± 2.94c	0.23 ± 0.01c	Hyperhydricity

The macronutrient component of the HZ medium was composed of NH_4_NO_3_, 520 mg⋅l^–1^; KNO_3_, 950 mg⋅l^–1^; KH_2_PO_4_, 495 mg⋅l^–1^; MgSO_4_⋅7H_2_O, 370 mg⋅l^–1^, and CaCl_2_⋅2H_2_O, 440 mg⋅l^–1^, with other mineral nutrients having the same composition as in MS. Different lowercase letters in the same column indicate a significant difference, as per Duncan’s multiple range test (p ≤ 0.05, n = 3 indicates three replicates).

**TABLE 3:** Effects of different concentrations and compositions of BA, NAA, and IBA on shoot induction, and range analysis.

No.	PGRs (μ M)			Shoot induction rate (%) (Mean ± SE; *n* = 3)	Shoot length (cm) (Mean ± SE; *n* = 3)	Growth state of shoots
	BA	NAA	IBA			
1	2.2	0	0	36.67 ± 1.93e	0.35 ± 0.01f	Thin shoots, short internode
2	2.2	0.5	0.5	43.33 ± 1.93d	0.28 ± 0.01g	Short shoots, large leaves
3	2.2	2.7	2.5	36.67 ± 1.93e	0.13 ± 0.00i	Small and short shoots
4	4.4	0	0.5	82.22 ± 1.11a	0.78 ± 0.00c	Robust shoots, fast growth
5	4.4	0.5	2.5	66.67 ± 1.93b	1.04 ± 0.02b	Robust shoots, small leaves
6	4.4	2.7	0	45.56 ± 1.11d	0.62 ± 0d	Thin shoots, callus
7	8.8	0	2.5	65.56 ± 1.11b	0.65 ± 0d	Robust shoots, small leaves, callus
8	8.8	0.5	0	57.78 ± 1.11c	1.12 ± 0.01a	Hyperhydricity
9	8.8	2.7	0.5	55.56 ± 1.11c	0.53 ± 0.01e	Hyperhydricity, plenty of callus
**Range analysis of induction rate**						
K_*BA*_0.9	38.89	K_*NAA*_0	61.48	K_*IBA*_0	46.67	
K_*BA*_4.4	64.82	K_*NAA*_0.5	55.93	K_*IBA*_0.5	60.37	
K_*BA*_8.8	59.63	K_*NAA*_2.7	45.93	K_*IBA*_2.5	56.30	
R_*BA*_	20.74	R_*NAA*_	15.55	R_*IBA*_	13.70	

Different lowercase letters in the same column indicate significant differences, as per Duncan’s multiple range test (p ≤ 0.05, n = 3 indicates three replicates).

### Shoot proliferation

The induced shoots were cut into approximately 1-cm sections and transferred into HZ medium. BA (0.9, 2.6 and 4.4 μM), NAA (0, 0.5 and 1.1 μM), and IBA (0, 0.5 and 1.0 μM), in different concentrations, were added to the medium. Nine treatments were designed according to the L9 (3^4^) orthogonal test (Table 4). After 7 weeks, the proliferation coefficient, number of effective shoots (≥0.5 cm) per explant, and growth state were recorded.

**TABLE 4:** Effects of different concentrations and compositions of BA, IBA, and NAA on shoot proliferation, and range analysis.

No.	PGRs (μ M)			Proliferation coefficient (Mean ± SE; *n* = 3)	Number of effective shoots per explants (≥ 0.5 cm) (Mean ± SE; *n* = 3)	Growth state of plantlets
	BA	IBA	NAA			
1	0.9	0	0	2.28 ± 0.01e	4.48 ± 0.09d	Little shoots, Unfolding leaves
2	0.9	0.5	0.5	2.25 ± 0.01e	4.43 ± 0.22d	Little shoots, Unfolding leaves
3	0.9	1.0	1.1	2.29 ± 0.04e	4.59 ± 0.14d	Folding Leaves, short shoots
4	2.6	0	0.5	2.75 ± 0.01c	5.32 ± 0.09bc	Unfolding leaves
5	2.6	0.5	1.1	2.71 ± 0.03c	5.21 ± 0.04c	Folding Leaves
6	2.6	1.0	0	3.00 ± 0.04b	5.63 ± 0.02b	Robust shoots, Unfolding leaves
7	4.4	0	1.1	2.95 ± 0.01b	5.60 ± 0.08b	Folding leaves
8	4.4	0.5	0	3.34 ± 0.02a	6.19 ± 0.18a	Short and small shoots
9	4.4	1.0	0.5	2.56 ± 0.04d	4.87 ± 0.08d	Folding leaves
**Range analysis of proliferation coefficient**						
K_*BA*_ 0.9	2.28	K_*IBA*_ 0	2.66	K_*NAA*_ 0	2.87	
K_*BA*_ 2.6	2.82	K_*IBA*_ 0.5	2.77	K_*NAA*_ 0.5	2.52	
K_*BA*_ 4.4	2.95	K_*IBA*_ 1.0	2.62	K_*NAA*_ 1.1	2.65	
R_*BA*_	0.67	R_*IBA*_	0.15	R_*NAA*_	0.35	

Different lowercase letters in the same column indicate significant differences, as per Duncan’s multiple range test (p ≤ 0.05, n = 3 indicates three replicates).

To reduce the browning rate, three anti-browning agents at different concentrations (Figure 1) were added to the proliferation medium for the experiment. There were a total of 10 treatments. Two groups of comparative experiments were set up. The first group was cultured directly under light conditions after inoculation, and the other group was cultured in the dark for 7 days and then transferred to a light culture. The browning rate was calculated after 14 days, and the proliferation coefficient was recorded after 7 weeks.

### Rooting

Robust shoots (2–3 cm in length) were harvested and transferred to half-strength HZ medium supplemented with different concentrations of NAA (1.1, 2.2, 3.2, 4.3, 5.4, and 8.1 μM) and IBA (1.0, 2.0, 2.9, 3.9, 4.9, and 7.4 μM) (Table 5). After 30 days of culture, rooting rate and growth state were recorded.

**TABLE 5:** Effects of different types and concentrations of auxin on rooting.

No.	Auxins	Concentration (μ M)	Rooting rate (%) (Mean ± SE; *n* = 3)	Average number of roots (Mean ± SE; *n* = 3)	Growth state of plantlets
1	NAA	0.0	20.00 ± 0.02f	1.43 ± 0.07e	Thin roots, callus less
2		1.1	50.00 ± 0.02cd	1.85 ± 0.04b	Stubby roots, callus less
3		2.2	52.22 ± 0.01c	1.83 ± 0.04bc	Stubby roots, callus less
4		3.2	83.33 ± 0.02a	2.19 ± 0.04a	Strong and long roots, callus less
5		4.3	78.89 ± 0.02a	2.09 ± 0.00a	Strong and long roots, callus more
6		5.4	70.00 ± 0.02b	1.90 ± 0.00b	Thin and short roots, callus more
7		8.1	73.33 ± 0.02b	1.94 ± 0.01b	Strong and long roots, callus more
8	IBA	1.0	54.44 ± 0.01c	1.73 ± 0.02cd	Thin and short roots, callus less
9		2.0	36.67 ± 0.02e	1.88 ± 0.06b	Thin and long roots, callus less
10		2.9	24.44 ± 0.01g	1.73 ± 0.01cd	Thin and long roots, callus less
11		3.9	45.56 ± 0.01d	1.66 ± 0.02d	Thin and long roots, callus less
12		4.9	36.67 ± 0.02e	1.66 ± 0.04d	Thin and short roots, callus more
13		7.4	34.44 ± 0.01e	1.68 ± 0.04d	Stubby roots, callus more

Different lowercase letters in the same column indicate significant differences, as per Duncan’s multiple range test (p ≤ 0.05, n = 3 indicates three replicates).

### Histology observation of differentiating root in microshoots

To investigate the rooting mechanism of *C. hystrix* plantlets, the formation of adventitious roots was observed by agarose sectioning. Starting from the induction of rooting culture, the bases of the 5–10 plantlets (0.5–1.0 cm in length) were cut and collected every 2 days and stored in a fixation medium [70% alcohol, acetic acid, and 38% formaldehyde (18: 1: 1 v: v: v)]. Then, the same volume of a mixture of TO (TO type biological tablet transparent agent) transparent agent and anhydrous ethanol, 30% anhydrous ethanol, and 70% TO transparent agent were used to treat the samples for 1 h. Finally, the samples were embedded in 3% agarose, cooled, solidified, and sliced with a vibratome (VT1000S, Leica, GER), with a thickness of 35 μm. They were then stained with toluidine blue, observed, and photographed using a microscope (BX43, Olympus, Japan).

### Acclimatization and transplantation

After 30 days of rooting culture, 1,000 plantlets with well-developed roots were placed in the greenhouse for 7–10 days. The plantlets were removed from the culture vessels, and the attached medium was washed away. Subsequently, the plantlets were transplanted to containers (containing a mixture of peat soil, perlite, and coconut bran 3: 1: 1 v: v: v). Before transplanting, the substrate was disinfected with potassium permanganate (800–1,000 ppm). Plantlets were watered thoroughly and kept in a greenhouse (25 ± 1°C, 80% RH, 70% shading). One week after transplanting, spray with 1% carbendazim and 2 weeks later, they were moved outdoors, and water and fertilizer (N: 10 g⋅l^–1^, P_2_O_5_: 10 g⋅l^–1^, K_2_O: 10 g⋅l^–1^, Fe: 0.05 g⋅l^–1^, Mn: 0.05 g⋅l^–1^, Nutrient solution and water 1:1,000 v: v) management were conducted daily. Survival rates were recorded after 30 days.

### Statistical analysis

The following formulas were used to calculate different plant regeneration parameters:

• Contamination rate (%) = number of contaminated explants/total number of explants × 100
• Browning rate (%) = number of browned explants/total number of explants × 100
• Survival rate (%) = number of surviving explants/total number of explants × 100
• Shoot sprouting rate (%) = number of sprouted explants/total number of explants × 100
• Proliferation coefficient = Total number of shoot ≥ 0.5 cm (in length)/Total number of regenerating explants
• Number of effective shoots per explant = total number of shoots ≥ 0.5 cm
• Rooting rate (%) = number of rooted explants/total number of explants × 100.
• Average root number per explant = total number of roots/numbers of rooted explants

All experiments were conducted in a completely randomized design with three replicates, and each treatment contained 30 explants, except for acclimatization. The results are presented as mean ± standard error (SE). The mean and SE values were calculated using Microsoft Excel 2019. IBM SPSS Statistics v26 (Armonk, NY, United States) was used for the statistical analyses. The significance of differences among the mean values was calculated using Duncan’s multiple range test at *p* ≤ 0.05. The results are presented as mean ± standard error of three replicates.

## Results

### Establishment of an aseptic system

Among the six treatments (Table 1), the best result was obtained with the combination of 1% (v: v) benzalkonium bromide for 2–4 min and 0.1% (w: v) mercuric chloride containing 2–3 drops of Tween 80 for 1–4 min. The survival rate of explants was up to 75%, and the contamination and browning rates were the lowest at 17 and 11%, respectively. This shows that multistage disinfection can significantly reduce the contamination rate of explants. This is consistent with the results of other researchers (Kuppusamy et al., 2019). Compared with treatment No. 5, the contamination rate of explants in treatment No. 4 without the addition of Tween 80 was significantly higher. Treatment No. 1, in which NaOCl alone was used as a disinfectant, had the highest contamination rate. The contamination rate of explants in treatments No. 3 and 6, in which 75% (v: v) ethanol was used, was significantly lower than that in treatment No. 1, but the browning rate was highest, at 25 and 27%, respectively.

### Shoot sprouting

Among the five media (Table 2), modified MS medium (HZ) was the optimal medium. We found that explants cultured on MS, 1/2 MS, and Gamborg’s B5 basal medium (B5) had a very low induction rate, and the induced shoots were short, thin and gradually died after 2–3 cycles of culturing. Shoots cultured on MS and B5 media showed hyperhydricity. The effect of shoot sprouting cultured on 1/4 MS was moderate; the shoots were thin and weak, and the stem segments were yellow-green. In contrast, the HZ medium positively influenced shoot sprouting; the shoot sprouting rate reached 71.11%, and the shoot length reached 0.95 cm. Moreover, the shoots grown on HZ medium were robust and green in color and elongated faster.

The results (Table 3) showed that different concentrations and combinations of the plant growth regulators (PGRs) significantly affected shoot sprouting. The optimal combination of PGR_*S*_ for *C. hystrix* was BA 4.4 μM and IBA 0.5 μM, wherein the shoot sprouting rate reached 82.22%, and the induced shoots were robust and grew rapidly (Figure 2C). In the culture medium containing 2.2 μM BA, the shoot sprouting rate was low, and the induced shoots were short. However, when BA reached 8.8 μM, hyperhydricity was observed in the shoots. Moreover, excessive auxin may lead to callus formation, in addition to inhibition of shoot sprouting.

### Shoot proliferation

The findings of this study suggest that different concentrations and ratios of PGRs (BA, IBA, and NAA) can promote the proliferation of *C. hystrix* plantlets to different degrees (Table 4). In the range of 0.9–4.4 μM of BA, the combination of BA and a single auxin can significantly improve the proliferation of plantlets. However, when the concentration of BA was 0.9 μM, the overall proliferation coefficient was the lowest. Range analysis showed that the influence of the three PGRs on shoot proliferation was BA > NAA > IBA. According to the range values, when the concentration of BA was 4.4 μM and that of IBA was 0.5 μM, the proliferation coefficient of shoots reached the highest (Figures 2D,E, 3.34). However, under these conditions, the cultured shoots were short, the leaves were folded, and fewer shoots were suitable for rooting. By contrast, the shoots cultured on a medium provided with BA (2.6 μM) and IBA (1.0 μM) were robust, the leaves were extended, and there were many shoots suitable for rooting, with the proliferation coefficient reaching 3.00 and the number of effective shoots reaching 5.63.

We observed that the proliferation coefficient of *C. hystrix* plantlets cultured in darkness for 7 days decreased slightly, but the browning rate was significantly lower compared with culturing directly under light after inoculation. Furthermore, the addition of vitamin C (VC), activated charcoal (AC), and polyvinyl pyrrolidone (PVP) at different concentrations affected the proliferation and browning of plantlets to different degrees (Figure 1). Compared with the control treatment (54.44%), the browning rate of plantlets was significantly reduced after adding anti-browning agents into the medium. VC showed the best anti-browning effect. When the concentration of VC was 80 mg⋅l^–1^, the browning rate of the plantlets was the lowest (13.33%) after dark treatment for 7 days, and there was a higher proliferation rate than in the control treatments. Under such culture conditions, the plantlets grew well, and the shoots were healthy. However, when the concentration of VC was 60 and 100 mg⋅l^–1^, the growth of the plantlets was poor. Different concentrations also reduced the browning rate and increased the proliferation rate. Under such culture conditions, plantlets generally grew with weak shoots and slow growth. AC had the worst anti-browning effect on *C. hystrix* plantlets. The addition of AC significantly reduced the proliferation coefficient, and most of the shoots died and showed poor growth.

### Rooting

The results showed that the addition of different concentrations of NAA and IBA significantly improved the rooting effect of *C. hystrix* plantlets compared with the control treatment (Table 5). The rooting rate first increased and then decreased with the increase in auxin concentration in treatments 2–7 and 8–13. We found that when auxin concentrations were too high, there were more calluses at the base of plantlets, which affected rooting quality. When auxin concentrations were too low, the roots were thin and the rooting rate was low. In terms of the rooting rate, rooting number, and growth state of plantlets, the overall rooting effect of NAA was better than that of IBA. Among them, when the concentration of NAA was 3.2 μM, the rooting rate was the highest (83.33%), the roots were strong, and the plantlets showed a substantial growth state (Figure 2F).

### Histology observation of differentiating root in microshoots

**FIGURE 3:**
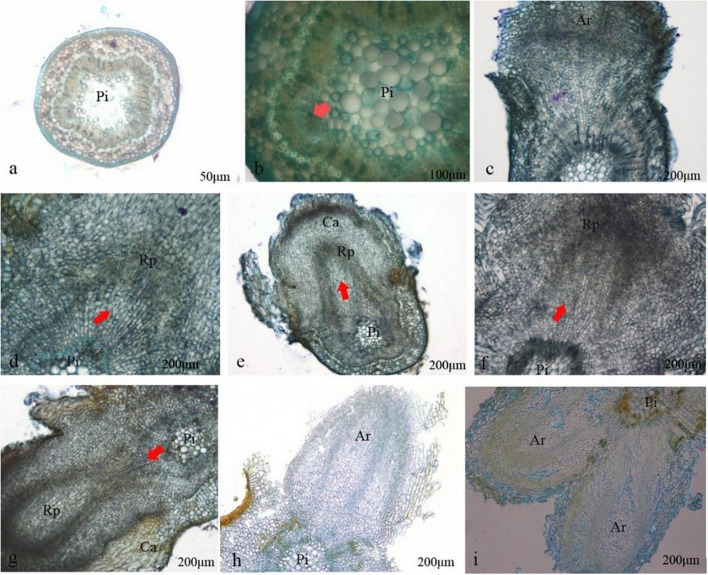
Histology observation on differentiating roots formation of *Castanopsis hystrix* plantlets. Ri, pith; Rp, root primordium; Ar, adventitious root; Ca, callus. **(a)** Rooted on day 4; **(b)** the cambium cells were active on day 5; **(c)** callus appeared; **(d)** root primordium appeared on day 7; **(e,f)** root primordium differentiated; **(g)** adventitious root broken through the cortex on day 11; **(h)** adventitious root broken through callus on days 12–13; **(i)** the adventitious root vascular connected to the stem vascular.

No significant change was observed after the *C. hystrix* plantlets were transferred into the rooting medium for 4 days. Starting from the day 5, the cambium outer cells showed periclinal division in succession and formed a ring band consisting of cells with thin walls, dense mass, and strong meristematic ability (Figures 3a,b). On day 6, local swelling at the base of the stem segment of the plantlets gradually formed calluses. These calluses were formed by resuming the division of cells surrounding the medullary rays (Figure 3c). On day 7, some active cells at the junction of the original cambium and medullary rays continued to divide into cell clusters deeper than other regions, forming the adventitious root primordium (Figure 3d). As the cells divided, the root primordium showed tissue differentiation after approximately 9 days. From the anatomical observation, the cells located at the morphological upper end of the root primordium gradually formed growth points and root caps, whereas the cells located at the lower morphological end of the root primordium were stained lighter, elongated, and formed oblong parenchymatous cells (Figures 3e,f). Around day 11, with the elongation, division, and differentiation of the root primordia cells, the root primordium penetrated the cortex and epidermis, grew out of the stem (Figure 3g), and then continued to penetrate the callus wrapped by the base of adventitious roots (Figure 3h), while the vascular cells differentiated from the adventitious root were connected to the vascular system of the stem segment (Figure 3i).

### Acclimation and transplantation

The regenerated plantlets transplanted into plastic cups had a survival rate of over 90% after 30 days, with bright green leaves and good growth (Figures 2G,H). The lignified plants that were transferred to the large container bags grew well. The regenerated plants were phenotypically identical to the parent plants and the leaves and dark green mature leaves.

## Discussion

The fuzziness of explants exposed outdoors for a long time is contaminated by various air pollution sources (Kang et al., 2020). In particular, adult trees in the wild have high levels of endophytic bacteria, resulting in tissue culture failure. Our preliminary experiments found that explants collected directly from mature trees in the field were heavily contaminated, making it difficult to establish an aseptic system. Therefore, we obtained a large amount of aseptic material by grafting superior genotypic branches as scions. Plants were cultivated in a greenhouse and regularly sprayed with 0.1% carbendazim. This approach has also been reported in other studies (Griebeler et al., 2014). The combination of Tween 80 and HgCl_2_ can also improve sterilization, and its superiority has been reported in previous studies (San-José et al., 2010; Martínez et al., 2017a). Our results are consistent with these findings.

During *in vitro* tissue culture, growth and morphology are mainly controlled by the composition of the basic culture medium (Carl and Richard, 2002; Kothari et al., 2004; Phillips and Garda, 2019). This study found that during shoot induction, except for culture on 1/4 MS and HZ, shoots induced from axillary shoots were shorter and gradually died after 2–3 cycles of culturing. It has been reported that inappropriate mineral nutrition in the medium, especially the proportion of macroelements such as N, P, and K, can affect the normal metabolism of plantlets (Nezami-Alanagh et al., 2019; Hameg et al., 2020). The experimental results showed that the low-salt medium is more conducive to the induction and growth of *C. hystrix*, which is the same as tissue culture research in *Quercus rubra* (Vengadesan and Pijut, 2009). High salt concentrations in the medium may cause hyperhydricity in plantlets (Oberschelp and Gonçalves, 2016; Nezami-Alanagh et al., 2018), which leads to chlorophyll deficiency in plantlet leaves, accumulation of water in intracellular spaces and walls, poorly developed cell walls, and reduction in cuticle thickness (Sen and Alikamanoglu, 2013; Tabart et al., 2015), as well as causes plantlets to exhibit a controlled emergency response with a phenomenon called hyperhydricity. Other nutrients can also affect plantlet growth. For example, Halloran and Adelberg (2011) found that nutrients such as Mg, Mn, and Ca affect the reproduction of *Curcuma longa*, and the effect of other elements on *C. hystrix* needs to be studied further. The optimization of a basic medium is a complex process; it can be optimized using computer technology, such as algorithm-based machine learning (artificial neural networks, fuzzy logic, and genetic algorithms) (Gago et al., 2010; Arteta et al., 2018; Nezami-Alanagh et al., 2018).

PGRs are important at all stages of plant tissue culture (Bidabadi and Jain, 2020) and are added to stimulate the formation of primordial roots in tissues presenting a rooting predisposition (Griebeler et al., 2014). In this study, BA had the most apparent effect on the induction and proliferation of *C. hystrix* shoots, confirming that BA plays a significant role in tissue culture (Namli et al., 2010). Cytokinins and auxins must be balanced during regeneration from explants (Skoog and Miller, 1957; Zhang et al., 2020); the concentration of PGRs was lower or higher and not conducive to the growth of shoots (Gonbad et al., 2014). The type and concentration of auxins are stricter in rooting cultures (Phillips and Garda, 2019). NAA was shown to stimulate rooting better than IBA, which is the opposite of *Panax ginseng* on rooting, indicating that the effect of auxins on roots varies from species to species (Kim et al., 2003). The combination of NAA and IBA has a better rooting effect (Khosh-Khui and Sink, 1982; Martínez et al., 2021); whether it applies to *C. hystrix* requires further verification.

The browning phenomenon is mainly caused by the action of oxidative enzymes, such as polyphenol oxidase (PPO) and phenylalanine ammonia lyase (PAL), with phenolic acid to produce toxic dark brown tissue culture plantlets (Shi et al., 2013; Zhou et al., 2018), leading to explant death and failure of regeneration (Tang and Newton, 2004; Zhenzhu et al., 2016; Shang et al., 2017). The factors affecting browning are complex and include the culture itself and the culture environment. VC was the best choice to inhibit the browning of *C. hystrix* plantlets, and darkness treatment for 7 days after inoculation resulted in a low browning rate. In contrast, PVP and AC had a negative effect on *C. hystrix* plantlets after culturing. This means that different types of anti-browning agents have different effects on the prevention of browning (González-Rodríguez et al., 2004). Another reason may be that PVP and AC act as adsorbents and compete with plantlets for nutrition in long-term culture (Cai et al., 2020). However, as an antioxidant, VC has less impact on plantlets and can even provide nutrition (Joy et al., 1988; Vijayalakshmi and Shourie, 2016). In addition, darkness treatments after inoculation can effectively reduce browning (Chavan et al., 2015; Serain et al., 2021) because proper dark handling increases endogenous phenol levels and the utilization of carbohydrates, which in turn leads to a higher rooting rate (Druart et al., 1982; Kang et al., 2020).

The root is an important organ for plants to absorb water and nutrients (Tanimoto et al., 1995). The formation of adventitious roots is a complex process controlled by many environmental and physiological factors (Deng et al., 2017), and it is essential to understand the formation and physiology of adventitious roots for breeding (Steffens and Rasmussen, 2016). In rooting cultures, plantlets are usually found at the base of hard-to-root plants, often called callus rooting. However, histological observation of adventitious roots showed that the root primordium of *C. hystrix* was produced from the interoperative formation layer, and no root primordia were formed in the callus, confirming that callus production was not directly related to the formation of root primordia. Some studies have shown that auxin-induced calluses share some characteristics with the root development pathway, which may promote the development of adventitious roots (Xu et al., 2018). Whether callus production promotes adventitious root growth in *C. hystrix* remains unknown. Once the callus has been proven to inhibit the growth of *C. hystrix* adnate roots, we can reduce the number of calluses during rooting by optimizing the rooting medium.

Previous studies have reported low survival rates of *Quercus rubra* during acclimation (Vengadesan and Pijut, 2009). However, in this study, more than 90% of the plantlets were preserved after acclimatization. According to their growth performance, these plantlets grew uniformly without excessive differences, showing a high degree of homogeneity between the regenerants. On the leaves, it was observed that the color of the regenerated plants was essentially the same as that of the parent leaves. Apical leaves were buff, whereas other leaves were dark green. Thus, *in vitro* propagation can maintain the excellent traits of the parent.

## Conclusion

This is the first report of the *in vitro* regeneration of mature *C. hystrix*. In this study, the obstacle of low survival rate of tissue culture of *C. hystrix* was successfully confirmed. We also solved other problems, such as the maximum number of shoots per explant was obtained on HZ containing 4.4 μM BA and 0.5 μM IBA, and markedly increased the shoot number and growth. For the optimal multiplication medium was on HZ containing 2.6 μM BA and 1.0 μM NAA, and for the rooting medium, the optimal hormone concentration was half-strength HZ + 3.2 μM NAA. The regenerated plants, which were propagated in this system, showed good growth, lush foliage, and well-developed roots. These results indicate that the regeneration protocol of *C. hystrix* can be used for clonal propagation of the superior genotype to lay the foundation for its popularization, preservation of germplasm, and genetic transformation.

## Data availability statement

The original contributions presented in the study are included in the article/supplementary material, further inquiries can be directed to the corresponding author/s.

## Author contributions

HZ: formal analysis, validation, and writing–original draft. MG: methodology and writing—original draft. QW, RL, and MZ: data curation and formal analysis. XD: supervision. RX: conceptualizations and resources, and writing–review and editing. All authors contributed to the article and approved the submitted version.
